# Acculturative Stress, Perceived Social Support, and Mental Health: The Mediating Effect of Negative Emotions Associated with Discrimination

**DOI:** 10.3390/ijerph192416522

**Published:** 2022-12-09

**Authors:** María José Rivera Baeza, Camila Salazar-Fernández, Diego Manríquez-Robles, Natalia Salinas-Oñate, Vanessa Smith-Castro

**Affiliations:** 1 Departamento de Psicología, Facultad de Ciencias de la Salud, Universidad Católica de Temuco, Temuco 4813302, Chile; maria.baeza@uct.cl (M.J.R.B.); dmanriquez2018@alu.uct.cl (D.M.-R.); 2 Laboratorio de Interacción, Cultura y Salud, Temuco 4813302, Chile; 3 Departamento de Análisis de Datos, Facultad de Ciencias Sociales y Humanidades, Universidad Autónoma de Chile, Temuco 4810101, Chile; 4 Departamento de Psicología, Facultad de Educación, Ciencias Sociales y Humanidades, Universidad de La Frontera, Temuco 4811322, Chile; natalia.salinas@ufrontera.cl; 5 Instituto de Investigaciones Psicológicas, Universidad de Costa Rica, San José 11501, Costa Rica; vanessa.smith@ucr.ac.cr

**Keywords:** migration, acculturative stress, perceived social support, mental health

## Abstract

The role of perceived social support in the acculturation process of immigrants remains unclear. In this study, we jointly evaluated the associations between acculturative stress and negative emotions associated with discrimination as antecedents of anxiety, depression, and stress symptoms in 283 immigrants living in Chile. Three competing models were tested via structural equation modelling to assess (1) the association among these variables and mental health symptoms and (2) to clarify the role of perceived social support. The third model was theoretically more adequate, showed a better fit, and explained 42.7% of the variance of mental health symptoms. In this model, perceived social support was associated with acculturative stress by reducing mental health symptomatology. Moreover, a direct relationship and an indirect relationship were found between acculturative stress (through negative emotions associated with discrimination) and mental health symptomatology. These results contribute to the understanding of the acculturation process experienced by immigrants in Chile and provide empirical evidence to be used to improve migration policies.

## 1. Introduction

Migratory processes have occurred since the dawn of humanity. However, in recent years, migration has experienced exponential growth due to globalisation [1]. Currently, there are more than 281 million international immigrants (hereinafter referred to as immigrant), a scenario far exceeding the forecast for the year 2050 by the International Organization for Migration (IOM), which predicted that there would be 230 million immigrants [2,3,4]. This trend continues in Latin America and the Caribbean with the so-called South–South migration; it is estimated that more than three million people have migrated to another country within the region, representing a larger number than South Americans residing in the United States or Spain (i.e., 2.7 million and 2 million, respectively) [5]. In this context, Chile has historically received an influx of diverse migrant groups given its social achievements (e.g., significant reduction of poverty) and economic and political stability, becoming an attractive place in the region to settle [6].

Chile’s immigrant population currently amounts to more than 1.6 million people, representing about 9% of the total population [2]. Immigrants are concentrated mainly in Santiago (national capital), and the northern macrozone of the country (main border with Bolivia and Perú) [7]. The increase in migration flows has generated a profound crisis at the national level, especially in the northern macrozone. High levels of xenophobia have characterised this crisis (e.g., antimigration protests and arson attacks on migrant camps), aggravated by the lack of adequate and up-to-date state policies (e.g., deportations) [8,9,10]. Because of this crisis in the northern macrozone, immigrants have had to look for settlement alternatives in the south of Chile [7,11,12]. This increase in migratory flows to new places of settlement in southern Chile highlights the need to understand how these acculturation processes affect the migrant population depending on the characteristics of the place of settlement, especially if these new settlements present particularities, such as intergroup conflicts and social vulnerability [13], considering that, in addition, Chile has stratified access to the health system and is one of the most unequal countries in the world [14,15].

The decision to migrate can be driven by diverse motivations, such as family reunification, search for greater well-being, better job, and economic opportunity, as well as those derived from sociopolitical conflicts in the countries of origin [12]. This process presents difficulties not limited to the mere arrival in the host country. However, it extends to the premigration stage, the journey, and the arrival and settlement in the host country [4,12,16]. Therefore, both the process and the conditions under which displacement takes place significantly impact the health of immigrants, as their quality of life and physical and mental health may be impaired [12,17,18,19,20,21].

### 1.1. Acculturative Stress, Emotions Associated with Discrimination, and Mental Health

In the adaptation process to the host country, immigrants must undergo various transformations at the intra- and interpersonal levels [22]. This socialisation process involves learning new guidelines, norms, and values and developing a new sense of social belonging [22,23]. However, the so-called acculturative stress occurs when the adaptation demands exceed the available resources [24,25]. Acculturative stress has been linked to mental health symptomatology in North America, Europe, Latin America, and Chile [26,27,28,29,30,31,32,33]. The literature has linked higher levels of acculturative stress with a higher prevalence of psychosomatic, depression, anxiety, and general psychiatric disorders [18,27,34,35,36,37]. Specifically, migrants in Chile have shown a high prevalence of acculturative stress. Studies have reported that migrants informed higher levels of nostalgia for their home country alongside experiences of discrimination, adaptation difficulties, and problems with their migratory regulation process, which implies several issues in obtaining access to health care, jobs, and housing, among others. Moreover, Chilean people struggle with the same concerns, which converts the migration to Chile into a highly challenging settlement [13,26,27]. Accordingly, this study hypothesises that, in immigrants, acculturative stress is positively related to mental health symptoms (H1).

One of the factors of acculturative stress that increases adaptation difficulties is the discrimination or unfair treatment by persons or groups from the host country (i.e., Chileans) [38,39]. These interactions are characterised by an unfavourable, uncontrollable, and threatening social evaluation that negatively impacts the immigrants. Thus, those who are discriminated against (i.e., immigrants) experience negative emotions, such as anger, sadness, uncertainty, and frustration, more frequently [40,41,42,43]. For their part, these negative emotions have been connected to various adverse mental health outcomes such as psychosomatic illnesses, distress, depression, anxiety, and stress [44,45,46,47,48,49]. On top of more frequently experiencing negative emotions associated with discrimination, immigrants must adjust the expression and modulation of these emotions to a cultural context different from that of their country of origin. The literature has pointed out that this emotional readjustment to the context is not without difficulties, as it is associated with increased acculturative stress, mental health symptoms, and psychosomatic symptoms, such as palpitations, tremors, and chest pains when breathing, among others [50,51,52,53,54]. This study hypothesises that there is a positive association between acculturative stress and negative emotions associated with discrimination (H2).

As for experiencing negative emotions, research on the indigenous population in southern Chile (i.e., the Mapuche people) has associated this type of emotion with adverse physical health outcomes [33,44,45,46,47,51,53,54]. Although this relationship has not been studied in other types of minority populations, such as migrants, descriptive evidence points to a high prevalence of negative emotions, such as anger, rage, and sadness, in the migrant population of southern Chile. This indicates that negative emotions could explain anxiety (i.e., as a mechanism), depression, and stress-related symptomatology [13,45,47,53,54]. Consequently, this study proposes a possible mediating role of emotions in the association between acculturative stress and mental health symptoms. In other words, we propose that acculturative stress in immigrants is positively associated with mental health problems because negative emotions are experienced (H3).

### 1.2. The Role of Perceived Social Support in Acculturative Stress and Mental Health Symptomatology

Social support is a multidimensional construct that includes several components such as instrumental or emotional support [55]. Social support can be perceived as coming from different sources such as family, friends, and significant others. In this way, perceived social support is defined as a set of interpersonal relationships that the individual perceives as sources of protection or affection, along with a sense of social belonging [56,57]. Consequently, perceived social support is a fundamental variable for immigrants because it facilitates their adaptation [58,59,60], mitigating the stressful consequences they experience during this process such as mental health problems [61,62,63,64,65,66,67]. However, the literature has also suggested that perceived social support can have a potential negative impact on the well-being of individuals. Some authors have shown that sources of alleged protection or affection can be perceived as adverse, unsolicited, or intrusive. Specifically, evidence has shown that when third parties aim to provide help or support, but their action oversteps or has no consent on the relationship boundaries, it could be harmful and threaten the recipient’s autonomy [68]. In this regard, qualitative and quantitative studies have suggested that perceived social support can be expressed on a continuum whose characteristics range from respectful to intrusive, directive, or overtly dominant [69]. As Chentsova [70] states, the perceived social support interpretation depends on cultural contexts because the same supportive act can be perceived as intrusive or friendly. Studies in individualistic settings have shown that perceived social support can provide emotional, interpersonal, and tangible resources but scaffold the recipient’s autonomy [71]. On the other hand, forms of unsolicited perceived social support (i.e., as a further imposition or highly directive help) are perceived with psychological costs to all parties, impacting the physical and mental health of the recipient and their well-being [72,73]. Moreover, this negative impact could be associated with various types of stress (e.g., interpersonal, psychological, and acculturative), mental health symptomatology, and adverse health outcomes [30,74]. The above suggests that the evidence regarding perceived social support is contradictory; therefore, it is essential to study the role of perceived social support [70], be it a barrier or facilitator, for adequate mental health in immigrants [75].

Perceived social support not only impacts mental health; several studies have shown that this variable also has a direct effect on acculturative stress, as having support networks facilitates the process of adaptation to a new culture [33,39,76,77,78], allowing for greater integration, bonding, and understanding of lifestyles, norms, and values [79,80]. Based on this information, this study hypothesises a direct and negative relationship between perceived social support and acculturative stress (H4). 

On the other hand, some research has reported that perceived social support has a positive effect on mental health by decreasing acculturative stress, whereas a lack of perceived social support may contribute to experiences of distress associated with negative emotions such as fear of exclusion [81,82,83]. Specifically, the literature has reported that perceived social support is associated with less depressive, anxiety, and stress-related symptomatology [61,84]. Conversely, the changes that occur during the migratory process regarding perceived social support (e.g., a decrease in the size of networks and their functionality) are associated with worse mental health indicators [27]. Thus, this study hypothesises a direct and negative relationship between perceived social support and mental health symptomatology (H5).

As discussed above, the effects of perceived social support on the reduction of mental health symptoms have been extensively documented; however, some studies suggest that the mechanism that could explain this reduction is mediated by the contribution of perceived social support to reduce the acculturative stress experienced by immigrants [79,85]. Thus, if people have perceived social support, their levels of acculturative stress should decrease, and they should report fewer symptoms of depression, anxiety, and stress [61]. Considering the above, acculturative stress is expected to mediate the relationship between perceived social support and mental health symptomatology (H5′).

### 1.3. Adaptation and Length of Residence in the Host Country

When adapting to the host country, various social, cultural, linguistic, and institutional difficulties impact the levels of acculturative stress [22,38,86]. In this regard, the evidence indicates that first-generation immigrants present more adaptation difficulties and worse levels of mental health symptomatology [87]. Furthermore, it has been observed that the longer the length of residence in the host country, the fewer the difficulties in adapting to sociocultural structures and functioning, which in turn results in lower rates of acculturative stress and fewer mental health symptoms [33,88]. However, the evidence in this regard is contradictory, as it has also been found that the passage of time has negative effects on the health status of immigrants [89,90], even reaching worse health levels than those of the host population [91,92].

A possible explanation for the above is the healthy immigrant phenomenon, which indicates that those who migrate are people who have better general health conditions, even better than those people with similar sociodemographic characteristics in the host country [93,94,95,96,97]. However, research conducted in North America, Europe, and Chile has revealed that the healthy immigrant phenomenon disappears over time, as with time, immigrants exhibit worse health indicators than the host population [23,89,90,98,99]. Another possible explanation is that there are two periods of increased mental health symptomatology in the migratory process. The first occurs immediately upon arrival in the host country, at which time the greatest adaptation difficulties occur; the second occurs 10 to 12 years after the arrival, once settlement has taken place and the person has made an assessment of the objectives and expectations regarding the process [100]. Therefore, since evidence indicates that the adaptation and length of residence affect acculturative stress, these variables will be controlled for in the present investigation.

### 1.4. The Present Study

Migration and its relationship to mental health is a growing phenomenon with significant consequences for migrants and host countries [2,4]. However, research in Latin America and the Caribbean, as well as in Chile, is scarce, with the latter focusing on the northern macrozone of the country and the national capital, despite the diverse economic, social, and cultural characteristics of both migrants and host cities [101]. This study aims to evaluate the migratory process in La Araucanía region, a multicultural area due to its high concentration of the Mapuche population, Chile’s largest indigenous people (33% of them reside in La Araucanía region) [102]. This area presents constant tensions in the intergroup relations between the Chilean State and the Mapuche people [103]. Given the above, it becomes interesting to investigate the migratory process in the context of conflicting intergroup relations.

**Figure 1:**
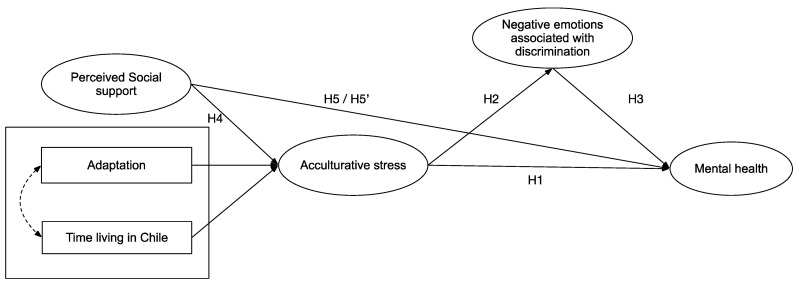
Proposed Model and Hypothesis.

Based on the conceptual model of Norbeck [104] and recent research performed by Luo and Sato [105], which proposed that acculturative stress has a mediating role between perceived social support and mental health symptomatology. Thus, this article aims to propose a model that comprehensively considers the role of perceived social support, adaptation, acculturative stress, and negative emotions associated with experiences of discrimination as predictors of mental health in Latino immigrants in La Araucanía region of Chile. Specifically, we hypothesised that acculturative stress has a direct and positive relationship to mental health symptoms (H1) and to negative emotions associated with discrimination (H2), such as anger, sadness, uncertainty, and frustration, which could act as mediators of this relationship (H3). On the other hand, a direct and negative relationship between perceived social support and acculturative stress is hypothesised (H4). Additionally, we will try to establish a direct relationship between perceived social support and mental health symptoms (H5), as well as an indirect relationship among these variables mediated by acculturative stress (H5′), to provide evidence contributing to the clarification of the contradictory results regarding the role of perceived social support in the literature. See Figure 1 for a graphic description of the study hypotheses.

## 2. Materials and Methods

The participants in the study consisted of 283 immigrants selected through convenience sampling conducted online. They had to meet the following inclusion criteria: be immigrants over 18 years of age, from Venezuela, Colombia, or Haiti, and residing in La Araucanía region. Those who were in the area for tourism were excluded. Their ages ranged from 18 to 72 years (*M* = 34.4, *SD* = 9.53). Of the participants, 83% were from Venezuela, 13% from Colombia, and 4% from Haiti. A further description of their characteristics is provided in Table 1.

### 2.1. Instruments

The participants had to answer a self-report questionnaire containing several sociodemographic variables and scales about their migration experience.

#### 2.1.1. Acculturative Stress

An ad hoc acculturative stress scale based on the Barcelona Immigration Stress Scale [106] and the Escala de Estrés para Inmigrantes [107] was used. This scale is composed of three factors: integration difficulties (four items, ω = 0.793), nostalgia (four items, ω = 0.690), and barriers (five items, ω = 0.764), which assess the immigrants’ acculturative stress levels in these dimensions. The participants had to respond to statements such as *I am affected by the differences between my culture and that of Chile*; *I miss the customs of my country*, and *We foreigners have worse working conditions* using a 5-point Likert-type scale (1 = *never* to 5 = *always*). Higher scores reflected higher levels of acculturative stress. The preliminary psychometric properties of this scale (manuscript under review) showed positive and significant associations (convergent validity) with mental health symptoms measured with DASS-21 [108].

#### 2.1.2. Negative Emotions and Affective States Associated with Discrimination

The scale from Baeza-Rivera [46] was used. It presents a bifactor structure that assesses the frequency with which immigrants experience negative emotions and affective states after experiencing a discrimination event related to their migratory process. The mobilising emotions subscale (e.g., *Helplessness* and *Anger*) is composed of seven items (ω = 0.931), and the passive emotions subscale (e.g., *Insecurity* and *Sadness*) is composed of nine (ω = 0.946). The participants had to answer using a 5-point Likert-type scale (1 = *not at all* to 5 = *very much*). Higher scores reflected an increased presence of negative emotions and affective states. This scale has demonstrated evidence of convergent validity based on its associations with health care behaviours [45].

#### 2.1.3. Mental Health Symptomatology

An ad hoc scale composed of three items was used to assess the frequency of depressive, anxiety, and stress symptoms immigrants may experience due to their migratory process. Specifically, people were asked to respond (e.g., *How often do you feel depressed, anxious, and/or stressed*) using a 7-point Likert-type scale measuring frequency (1 = *never* to 5 = *very often*). Higher scores reflected an increased presence of this type of symptomatology (ω = 0.870). This ad hoc scale showed positive and significant associations with the Depression, Anxiety, and Stress Scale—21 (*r* = 0.585, *p* < 0.001), so its use was preferred due to its simplicity.

#### 2.1.4. Perceived Social Support

An ad hoc perceived social support scale based on the Multidimensional Scale of Perceived Social Support [56] assessing the sources of perceived social support was used to measure the frequency of perceived social support in the month prior to the interview. The scale is composed of four items (ω = 0.783). Participants responded (e.g., *My colleagues try to help me; I can count on my colleagues when things go wrong; I can talk about my problems with my colleagues*) using a 5-point Likert-type scale (1 = *never* to 5 = *always*). Higher scores reflected higher perceived social support.

#### 2.1.5. Control Variables

The effect of the length of residency in Chile and adaptation difficulties was controlled. The participants had to answer how long they had been residing in the country in months (see Table 1). In addition, they answered a question aimed at assessing adaptation difficulties (e.g., *How hard has it been for you to adapt?*) using a 7-point Likert-type scale (1 = *not at all* to 7 = *very much*). Higher scores reflected greater adaptation difficulties.

### 2.2. Data Collection

The Ethics Committee of the authors’ affiliation university approved this study (Res. N° 21/18). The survey was applied through Google Forms online from July to November 2021. This format allowed access to participants while reducing the risk of contagion of COVID-19. It included an informed consent form that indicated the study’s objective, ensured anonymity and confidentiality, and provided the contact details of the responsible researchers. Participation in the study was remunerated by approximately USD 10. Answering the survey took approximately 15 min.

### 2.3. Data Analysis

Data were explored at a descriptive level, finding that asymmetry and kurtosis were acceptable. Considering the data’s ordinal nature, we performed Spearman correlations to test the association among the study variables. We used McDonald’s omega to evaluate the scales’ internal consistency [109]. Structural equation models were estimated to test the proposed relationship pattern using the *lavaan* package [110] of the *R* software [111]. We used the diagonally weighted least square method, which was more suitable for ordinal data [112], to estimate our models. As most of the scales used in this study were large, although they reported appropriate internal consistency values, we decided to calculate the means of each factor of the scale and model them as indicators of our latent variables. That said, negative emotions associated with discrimination were modelled using two indicators representing the mean of the passive and catalytic emotions scale. Acculturative stress was specified by three indicators: a mean of the factor of integration difficulties, nostalgia, and barriers. We specified a model in which perceived social support, as a latent variable, consisted of four items and mental health consisted of three items (see items in the instruments section).

The models were evaluated using the conventional fit indices: χ^2^, the comparative fit index (CFI), the Tucker–Lewis index (TLI), the square root of the mean error of approximation (RMSEA) with its confidence interval at 90%, and the square root of the standardised mean residuals (SRMR). These indices were interpreted using conservative goodness-of-fit criteria: CFI and TLI > 0.95 and RMSEA and SRMR ≤ 0.06 [113]. For comparison among models, we used the ΔCFI, as Cheung and Rensvold [101] stated that the chi-square difference test is not reliable due to sampling sensitivity. Cheung and Rensvold [114] proposed that models are no different if the ΔCFI is less than 0.010. Finally, we evaluated the indirect effects of acculturative stress on mental health through negative emotions associated with discrimination.

## 3. Results

The correlations among the study variables and their respective descriptive data are shown in Table 2.

**Figure 2:**
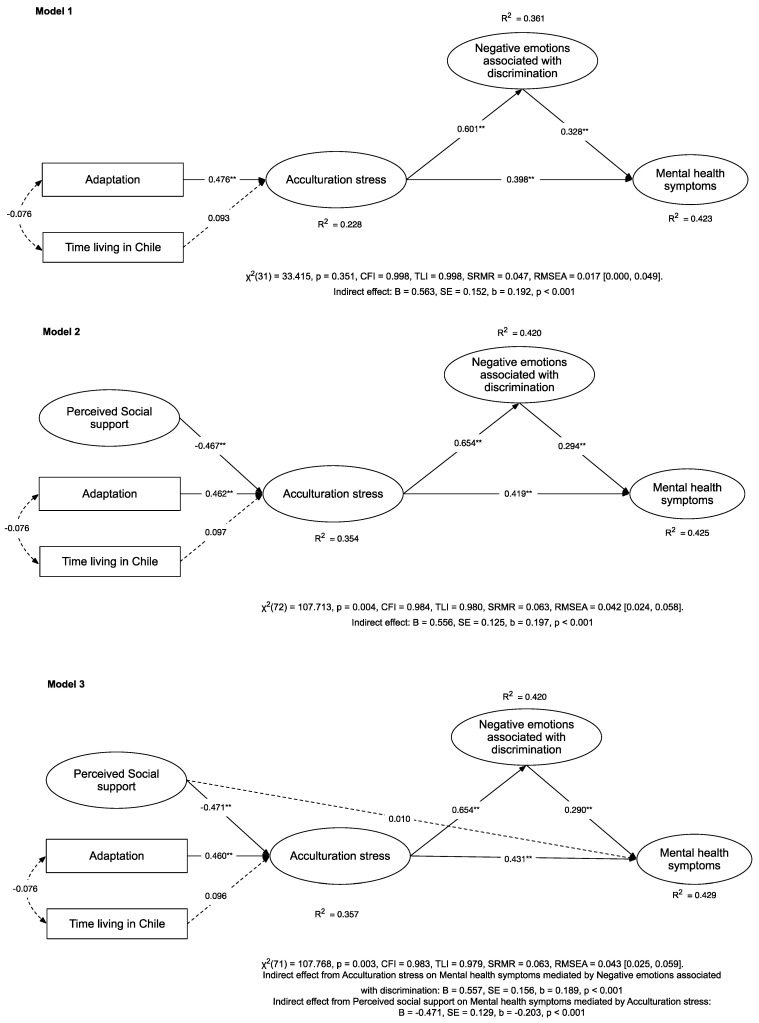
Models tested: 1, 2, and 3. ** *p* < 0.001.

Following the proposed relationships established in the introduction, we tested a model evaluating mental health (symptoms of anxiety, depression, and stress) predicted by acculturative stress (H1) and mediated by negative emotions associated with discrimination (H2 and H3). This model showed an excellent fit (see Table 3, Model 1). The proposed model of relationships explained 41.5% of the mental health symptoms experienced by immigrants in Chile, 36.1% of the variance of negative emotions associated with discrimination, and 22.8% of the variance of acculturative stress (see Figure 2, Model 1). This model found a positive and direct association between acculturative stress and mental health and an indirect effect of acculturative stress on mental health through the negative emotions associated with discrimination (B = 0.563, SE = 0.152, β = 0.192, and *p* < 0.001). These results confirm H1, H2, and H3. Moreover, this partially mediated effect represented 31.4% of the total effect of acculturative stress on the mental health of immigrants.

As hypothesised (H4), we proceeded to test the association between perceived social support and acculturative stress by adding this parameter to the previous model (see Figure 2, Model 2). This model also showed an excellent fit (see Model 2 in Table 3). As in Model 1, we also found an indirect effect of acculturative stress on mental health through negative emotions associated with discrimination (B = 0.556, SE = 0.125, β = 0.197, and *p* < 0.001). This partially mediated effect represented 33.1% of the total effect of acculturative stress on mental health (an increase of 1.7%). Model 2 explained 0.04% more of the variance of mental health (total variance = 42.9%), 5.9% more of the variance of negative emotions associated with discrimination (total variance = 36.1%), and 12.6% more of the variance of acculturative stress (total variance = 35.4%). On a more critical note, in this model, and as expected (H4), we found a negative association between perceived social support and acculturative stress. This association means that higher levels of perceived social support are associated with lower levels of acculturative stress.

Finally, as hypothesised, we decided to add a direct path from perceived social support to mental health (H5) and test whether there was an indirect effect through acculturative stress (H5′). This parameter led to estimating a new model (See Model 3 in Figure 2) (We estimated this model controlling for individual characteristics of migrants, such as educational level, monthly income, sex, and country of origin, and we did not find statistically significant differences with Model 3, Δχ^2^ = 53,759 (df = 40), *p* = 0.070, ΔCFI = 0.05, and Δ RMSEA = 0.03). This model also showed an excellent fit (See Model 3 in Table 2). Model 3 explained 42.7% of mental health symptoms experienced by immigrants in Chile, 0.02% more than Model 2. Interestingly, when comparing Model 3 with Model 2, the former explained 6.7% more of the variance of negative emotions associated with discrimination (total variance = 42.8%) and 8.2% more of the variance of acculturative stress. In Model 3, we replicated the indirect effect of acculturative stress on mental health through negative emotions associated with discrimination (B = 0.557, SE = 0.156, β = 0.189, and *p* < 0.001), as in the previous models. This partially mediated effect represented 30.6% of the total effect of acculturative stress on the mental health of immigrants. The path from perceived social support to mental health was not significant (β = 0.010 and *p* = 0.851). As there was no direct effect of perceived social support on mental health, we decided to test an indirect effect through acculturative stress. We found that this indirect effect was significant (B = −0.471, SE = 0.129, β = −0.203, and *p* < 0.001) and that all the effect of perceived social support was explained through acculturative stress (indirect effect represents 100% of the total effect). Thus, we did not find support in this model for the direct effect of perceived social support on mental health (H5), but rather for the indirect effect of perceived social support on mental health through acculturative stress (H5′).

## 4. Discussion

This article aims to propose a model that comprehensively considers the role of perceived social support, adaptation, acculturative stress, and negative emotions associated with experiences of discrimination as predictors of mental health in Latino immigrants in a relatively understudied context, i.e., La Araucanía, Chile. For this purpose, three models that evaluated these antecedents and their relationship with mental health symptomatology were tested, controlling for the effect of acculturative stress on the length of residence and the level of adaptation to the host country (Chile). In this regard, the results reveal that the three models present adequate fit indicators. However, in addition to explaining a higher percentage of the variance of mental health symptomatology (42.7%), the third model allowed us to clarify the role of perceived social support as an antecedent of mental health symptomatology through its effect on acculturative stress.

In general, we found in the sample medium high levels of self-reported mental health symptomatology, with 45.6% of the participants reporting symptoms of depression, 61.5% of anxiety, and 72.1% of stress. This is consistent with the evidence that migratory processes are associated with a higher incidence of mental health symptoms, such as depression, anxiety, and stress, as well as other types of psychiatric disorders [24,27,87,91,115,116]. The high presence of these negative symptoms is likely due to the demands of the process and the challenges of achieving an adequate adaptation to the host country [22,25]. A possible explanation for the present study is that, since the participants are first-generation immigrants, they are exposed to more difficulties than second- or third-generation immigrants [87,117]. For example, for first-generation migrants, it is more challenging to settle in a new context where they have less formal and informal support. Moreover, this is more complex when settling into a new environment with adverse weather conditions for these migrants in La Araucanía (cold and rainy weather, which is extremely different from the Ecuadorian region where they come from). Future studies should evaluate whether this phenomenon occurs in families that migrate frequently or belong to the second or third generations in the host country and the consequences of coming from such families.

The results presented in this article reveal high levels of mental health symptomatology in immigrants [20,27], which contrasts with the healthy immigrant phenomenon described in the literature. According to Holz [94], immigrants are reported to have a better general health status than the host population upon arrival in the host country [93,94,95,96]. However, these studies focus on aspects associated with physical health, which cannot be equated with the complex aspects of mental health such as those considered in this study.

Regarding acculturative stress, evidence suggests it is one of the main predictors of mental health indicators [32]. In this regard, the results of this study indicate that higher levels of acculturative stress are associated with higher levels of mental health symptomatology (*β* = 0.433 and *p* < 0.001). This shows that the migration aspects that generate stress for immigrants, such as barriers to integration, nostalgia for one’s customs, and significance of people staying in their country of origin, as well as difficulties generated by experiences of discrimination, take a significant psychological toll on the immigrants’ mental health [27,29].

A vital aspect of the model proposed is emotions, as they constitute a psychological element that influences health behaviours and outcomes [47,48]. In this study, negative emotions associated with discrimination, such as anger, sadness, uncertainty, and frustration, are directly related to increased mental health symptomatology in immigrants. These findings are consistent with recent research that give emotions a prominent role in predicting health outcomes and behaviours [42,45,46,53]. The above is even more relevant when considering that experiencing situations of discrimination and the consequent emotional response hinders integration, as the interactions established between immigrants and persons or groups from the host country are affected, which in turn modulates future behaviours between both groups and the limits or parameters for the expression of emotions [118]. Therefore, experiencing positive emotions promotes healthy interpersonal relationships, which contribute to greater adaptation without generating high cognitive demands or mental health costs [119].

Emotions are not only directly related to mental health symptomatology, but according to this study’s findings, they mediate between acculturative stress and mental health (*β_indirect_* = 0.557 and *p* < 0.001). Thus, the experience of negative emotions resulting from discrimination is greater when acculturative stress is experienced, which leads to increased mental health symptomatology. Therefore, it is essential to study the role played by emotions during acculturation processes, as although positive emotions can promote greater cultural integration and successful functioning in different societies, experiencing negative emotions associated with acculturative stress hinders the effective use of cognitive resources and coping strategies available to deal adaptively with experiences of discrimination, and thus mental health symptomatology increases [119].

Evidence suggests that the role of perceived social support in mental health symptomatology is contradictory. In this regard, the results of this study provide relevant empirical evidence to clarify the paradox existing in the literature concerning the role of perceived social support as a barrier or facilitator of mental health in immigrants. To be specific, we found that there is no significant direct relationship between perceived social support and mental health symptomatology; however, an indirect effect was observed through acculturative stress. This absence of a direct effect implies that the perception of social support does not affect mental health symptomatology. Moreover, an indirect effect implies that the perception of social support that diminished the acculturative stress (i.e., as a mechanism) lessens mental health symptomatology. We acknowledge that the variable measured was perceived social support and that it has limitations because it does not inform about the number of support networks nor if the support comes from the country of origin or the host country. Future studies should address these limitations and assess not only the perception of social support but the origin and amount of it. Specifically, we found that perceived social support does not play a role by itself in the levels of mental health symptomatology but that its effect is mediated by social, cultural, linguistic, and institutional difficulties that impact the levels of adaptation and integration to the host country, affecting acculturative stress and, thereby, mental health symptomatology [58,120]. Therefore, we observed that perceived social support mitigates mental health symptomatology by decreasing acculturative stress and its consequences on mental health. In this study, we found that, without considering perceived social support, acculturative stress is explained by 22.8% (Model 1), whereas when perceived social support is included (Model 3), acculturative stress is explained by 35%. That said, perceived social support contributes to explaining and understanding acculturative stress in immigrants. Future research should also address the emotions that are associated with perceived social support. Exploring this topic could lead to a promising explanatory process.

The present study provides relevant evidence on the role of various variables, such as perceived social support, acculturative stress, and negative emotions, associated with discrimination in immigrant mental health symptomatology levels. The background information we gathered contributes to the design of evidence-based strategies to promote the adaptation of immigrants to new host cultures and societies as well as to guide and facilitate the provision of the assistance required during this process, reducing the costs that migration can bring both at the individual and social levels for the host country [12,101,121]. Similarly, the results of this model reveal that reducing discrimination and integration barriers would, in turn, reduce acculturative stress. Hence, public policies should focus on promoting sociocultural integration processes (e.g., simplifying the bureaucracy of migration laws and regulations and/or facilitating access to fundamental rights such as health care, education, housing, work, and social security) [28,122,123]. These public policies, especially in terms of health, must consider the characteristics of the migrant population, both those associated with the self-reporting of individual health statuses and those linked to the health systems of their countries of origin, since the information that migrants deliver to health professionals in Chile may not be consistent with their current health status, or it may be understood differently due to cultural differences. In addition, it is essential to promote social interactions aimed at integration and good treatment, which will help reduce stereotypes, prejudices, and discriminatory behaviours toward immigrants. In this way, the adaptation process will be less complex, promoting the experience of positive emotions, which will reduce mental health symptomatology.

The present research constitutes a valuable contribution to understanding migration and its implications for mental health. However, some limitations should be considered. One limitation is the fact that this is a cross-sectional study. Future research should consider using longitudinal panel designs to evaluate the temporal stability of the relationships presented in this article. Another limitation of the study is data collection, given that it was conducted online due to the confinement during the COVID-19 pandemic in Chile. Data collection considerably limited the number of participants as there was a lack of confidence on the part of migrants to provide information regarding their migratory process for fear that it could be misused. This is relevant because immigrants in Chile have experienced several situations of deception and misuse of data that have undermined their trust in Chileans (e.g., in the workplace and deportations). Therefore, giving personal information online might have undermined the data collection process (by preventing access to a larger sample) and made self-selection bias present. Thus, the immigrants who participated probably had the necessary documentation and access to the internet and were willing to give their information, even though all of them were assured that the study complied with all the ethical safeguards. Future studies should seek possible solutions to this problem to make the results more generalisable.

Based on the reported findings, we suggest that future lines of research consider aspects such as the conceptualisation of mental health symptoms, which may vary across cultures both in their understanding and expression; knowing how they are understood and expressed may contribute to the understanding of mental health. In this study, we assessed mental health symptoms to avoid cultural bias associated with understanding and conceptualizing mental health problems. Extensive research has provided evidence that language and cultural adaptions are potential barriers to access health systems and the quality of health care attention, impacting mental health problems [124]. Future research should consider how mental health problems are conceptualized and the meaning different migrant populations attribute to them, following the guidelines of the American Psychological Association to establish culturally relevant diagnoses and treatments. Another element to consider is related to continuing to explore the role of perceived social support in the mental health outcomes of immigrants. Although this study provides relevant evidence in this regard, further research in more heterogeneous samples is required, clarifying the mechanisms by which it becomes a protective factor and those by which it constitutes a risk factor as the positive or negative effect of perceived social support is not only determined by the absence or presence of support networks but, more importantly, by the quality of the sources that provide perceived social support, as well as the types of support received, whether instrumental or emotional [125]. Future studies on acculturation should also consider the importance and benefits of incorporating control variables, such as those included in the present study, when evaluating complex hypotheses with cross-sectional data.

Finally, the findings of this research should be interpreted with caution because the migratory process is contextually dependent. This means that migration is specific to the characteristic of the migrant groups (sociodemographic, migration motives, and others) and the characteristics of the host culture (discrimination levels toward migrants, migration politics, health conditions for the migrant population, and migrant population). Moreover, this should be considered when considering the intergroup conflict context and higher levels of multidimensional poverty and inequity of La Araucanía region in Chile. Despite this, these results are a relevant input to understanding the relationship between migration and mental health in a particular population that migrates to Chile.

## 5. Conclusions

Migration represents a phenomenon with significant consequences for immigrants and host countries; therefore, understanding the relationships among the variables that influence mental health provides background information that helps to understand the phenomenon better and to guide future efforts in terms of public policies promoting and facilitating the integration of immigrants in the host countries.

## Figures and Tables

**Table 1:** Sociodemographic characteristics of the sample.

	*n* (%)
Age	
Mean (standard deviation)	34.40 (9.53)
Country of origin	
Colombia	36 (13%)
Venezuela	235 (83%)
Haiti	12 (4%)
Marital status	
Single	135 (47%)
Married	69 (24%)
Divorced	7 (2%)
De facto separated	8 (3%)
Cohabiting	64 (23%)
Sex	
Female	189 (67%)
Male	94 (33%)
Socioeconomic status	
Low, very low	158 (55%)
Medium	120 (42%)
High, very high	5 (3%)
Level of education	
Complete secondary education or below	84 (30%)
Complete or incomplete undergraduate education	177 (62%)
Postgraduate education	22 (8%)

**Table 2:** Spearman correlations among the study variables.

	1	2	3	4	5	6
1. Time living in Chile	-					
2. Adaptation difficulties	−0.084	-				
3. Perceived social support	−0.037	−0.175 *	-			
4. Acculturative stress	−0.033	0.389 *	−0.248 *	-		
5. Negative emotions associated with discrimination	0.137 *	0.254 *	−0.259 *	0.460 *	-	
6. Mental health symptoms	−0.040	0.265 *	−0.220	0.423 *	0.502 *	-
Mean (SD)	32.131 (22.460)	3.795 (1.530)	3.330 (1.020)	2.480 (0.658)	2.880 (0.840)	2.424 (0.957)

Note. * *p* < 0.05.

**Table 3:** Fit indices of the models tested.

Model	$\chi^{2}$	*df*	CFI	TLI	RMSEA (90% CI)	SRMR	Model Comparison	∆CFI
Model 1	33.415	31	0.998	0.998	0.017 (0.000, 0.049)	0.047	-	-
Model 2	107.713 *	72	0.984	0.980	0.042 (0.024, 0.058)	0.063	Model 2 vs. Model 1	−0.014
Model 3	107.768 *	71	0.983	0.979	0.043 (0.025, 0.059)	0.063	Model 3 vs. Model 2	−0.001

Note. * *p* < 0.05.

## Data Availability

Not applicable.
